# Trends in antenatal care attendance and health facility delivery following community and health facility systems strengthening interventions in Northern Uganda

**DOI:** 10.1186/1471-2393-13-189

**Published:** 2013-10-18

**Authors:** Michael Ediau, Rhoda K Wanyenze, Simba Machingaidze, George Otim, Alex Olwedo, Robert Iriso, Nazarius M Tumwesigye

**Affiliations:** 1 ChildFund Uganda, P.O Box 3341, Kampala, Uganda; 2 Makerere University School of Public Health - CDC Fellowship Program, P.O Box 7072, Kampala, Uganda; 3 Kitgum District Local Government, Directorate of Health Services, P.O Box 28, Kitgum, Uganda; 4 Makerere University School of Public Health, P.O Box 7072, Kampala, Uganda; 5 Baylor College of Medicine Children’s Foundation Uganda, P.O Box 72052, Kampala, Uganda

**Keywords:** Trends, Antenatal care, Health facility delivery, Community, Health facility, Systems strengthening

## Abstract

**Background:**

Maternal morbidity and mortality remains high in Uganda; largely due to inadequate antenatal care (ANC), low skilled deliveries and poor quality of other maternal health services. In order to address both the demand and quality of ANC and skilled deliveries, we introduced community mobilization and health facility capacity strengthening interventions.

**Methods:**

Interventions were introduced between January 2010 and September 2011. These included: training health workers, provision of medical supplies, community mobilization using village health teams, music dance and drama groups and male partner access clubs. These activities were implemented at Kitgum Matidi health center III and its catchment area. Routinely collected health facility data on selected outcomes in the year preceding the interventions and after 21 months of implementation of the interventions was reviewed. Trend analysis was performed using excel and statistical significance testing was performed using EPINFO StatCal option.

**Results:**

The number of pregnant women attending the first ANC visit significantly increased from 114 to 150 in the first and fourth quarter of 2010 (OR 1.72; 95% CI 1.39–2.12) and to 202 in the third quarter of 2011(OR 11.41; 95% CI 7.97–16.34). The number of pregnant women counselled, tested and given results for HIV during the first ANC attendance significantly rose from 92 (80.7%) to 146 (97.3%) in the first and fourth quarter of 2010 and then to 201 (99.5%) in the third quarter of 2011. The number of male partners counseled, tested and given results together with their wives at first ANC visit rose from 13 (16.7%) in the fourth quarter of 2009 to 130 (89%) in the fourth quarter of 2010 and to 180 (89.6%) in the third quarter of 2011. There was a significant rise in the number of pregnant women delivering in the health facility with provision of mama-kits (delivery kits), from 74 (55.2%) to 149 (99.3%) in the second and fourth quarter of 2010.

**Conclusions:**

Combined community and facility systems strengthening interventions led to increased first ANC visits by women and their partners, and health facility deliveries. Interventions aimed at increasing uptake of maternal health services should address both the demand and availability of quality services.

## Background

Globally, it is estimated that 342 900 (uncertainty interval 302 100–394 300) maternal deaths occurred worldwide in 2008 [1]. Progress towards achievement of millennium development goal (MDG) 5 has been slow [1-3] with only 23 countries reported to be on track to achieve a 75% decrease in maternal mortality rate (MMR) by 2015 [1]. Skilled delivery care is considered a crucial function within the health care system for saving the lives of mothers and newborns and is an important indicator for monitoring MDG 5 [4]. Increasing the coverage of skilled delivery care and ANC depends upon improved training and monitoring of health care providers, and greater family participation in ANC visits [5].

ANC visits constitute one of the few times in which women in many resource-poor settings seek care for their own health [6] and, represents an important opportunity to identify and treat problems such as anaemia and infections and for prevention services like prevention of mother to child transmission of HIV (PMTCT), help women best prepare for birth, as well as inform them about pregnancy-related complications, and the advantages of skilled delivery care with at least four ANC visits being recommended for a normal pregnancy [7-9]. In a rural district hospital setting in Malawi, at least 90% of mothers attending antenatal services accepted HIV voluntary counselling and testing (VCT), of whom approximately one-quarter were HIV-positive and enrolled into the PMTCT programme [10]. Women who attend ANC are also more likely to seek skilled delivery care [11-14]. Nevertheless, 20% of all women who attend ANC four times or more in sub-Saharan Africa, do not seek skilled delivery attendance [13].

Women’s preferences for a home birth and lack of planning for delivery are reinforced by the failure of health care providers to consistently communicate the importance of skilled delivery and immediate post-partum care for all women during routine antenatal visits [5]. In a rural district in Tanzania, deliveries with a skilled attendant increased from 34.1% to 51.4% following engagement of community based safe motherhood promoters (SMPs). This improvement was attributed to the home visits by SMPs, the close collaboration with existing community structures and health services as well as targeting of individual women and involvement of influential people within the communities [15].

Spouses are likely to exert strong influence on the attitude of pregnant women towards VCT during ANC [16]. Men generally do not accompany their wives for ANC and are not present in the labour room during delivery [17]. However, men are socially and economically dominant and are gatekeepers of women’s reproductive health. They make decisions about the timing and conditions of sexual relations, family size, and access to health care including ANC attendance and place of delivery [5,18]. This situation makes men critical partners for the improvement of maternal health and reduction of maternal mortality. Male partners who attend ANC are also likely to accept HIV testing [19]. Thus, there is a need to increase involvement of men in their partner’s maternity care. Although male involvement remains a challenge, some categories of men like; the educated, younger husbands, those in monogamous marriages and non Muslims have seen to achieve this and are able to accompany their wives for ANC services [18]. Identifying and training a critical mass of these “change agents” may increase male involvement [18]. Empowering male partners with knowledge about ANC services may also increase their ANC participation and in turn increase skilled attended deliveries by their pregnant wives [20]. Raising the awareness of men and engaging them in birth preparedness and complication readiness enables their support for early spousal utilization of these services [4]. Similarly, preparing for birth and being ready for any possible pregnancy complications could reduce all three phases of delay and thereby positively impact birth outcomes [21]. Barriers to male partner involvement include complex and interrelated structural and cultural issues and require community sensitization as well as improving client-friendliness in the clinics in order to mitigate the social and cultural factors [22].

In Uganda, a number of organizations have implemented various interventions to improve access to maternal health services including ANC and skilled delivery attendance. However, these programs have focused either on raising demand for services or strengthening of health facilities to improve the quality of services, and have not addressed both of these issues concurrently. Documentation of successes and failures of such interventions is also limited. This program evaluation aimed at documenting trends in key ANC and delivery indicators following implementation of program interventions to simultaneously increase demand for, and utilization of services as well as address quality gaps at a health center in rural Uganda.

## Methods

### Study area

The interventions were conducted in Kitgum Matidi Sub County in Kitgum District, Northern Uganda. Kitgum Matidi (HC) III serves as the highest level health facility providing health care services in Kitgum Matidi Sub County. The facility serves an estimated population of about 15,000 people. The health facility provides a range of preventive and curative health services including: ANC, skilled attended delivery, other outpatient and inpatient services.

### Description of program interventions

The project interventions focused on strengthening both the community and health facility systems in order to increase demand and quality of services, and ultimately utilization of ANC and skilled delivery services at the health facility. The project interventions were implemented between January, 2010 and September, 2011. The interventions mainly targeted pregnant women and their male partners. Details of the program interventions are described below. The key components of the interventions are also summarized in Table 1.

**Table 1:** Summary of selected program interventions implemented

**Selected interventions implemented**	**By who**	**Method of delivery**	**Duration of intervention**	**Frequency of intervention**	**Target group**
**Community based interventions**					
Training of 60 VHTs and provision of bicycles and sensitization job aids (charts)	Project team	Training was conducted in 2010 and a refresher training conducted in 2011	5 days training in 2010 (training) and 3 days in 2011 (refresher)	Twice (once per year)	VHTs
Community mobilization and sensitization on ANC and skilled (health facility) delivery attendance as well as PMTCT	VHTs	Community meetings	21 months (entire project period reported in the paper)	At least weekly	Pregnant women(and other women of child bearing age) and men
Identification and orientation of male partner ANC/PMTCT access clubs on their roles	Project team	Identification was done through local leaders and health workers’ recommendation based on exemplary behaviour. Orientation was done through meetings.	2 weeks for identification and 3 days for orientation	Once	Men with exemplary behaviour in ANC/PMTCT attendance
Dialogue and mobilization meetings	Male partner ANC/PMTCT access clubs	Community meetings with other men	21 months	Twice every month	Men in the community
Community mobilization and sensitization on ANC	MDD groups	Staging MDD performances in public places like markets among others	21 months	At least twice every month	Pregnant women (and other women of child bearing age) and men
Follow-up and referral of pregnant women and their spouses to attend ANC and deliver in health facilities	VHTs	Home visits	21 months	Routinely as need arose	Pregnant women and their spouses
Strengthening the linkage between the community and health facility	VHTs and health workers	Performance review meetings between VHTs and health workers. In these meetings, health workers also provide support supervision to VHTs	20 months	Monthly	VHTs and health workers
Holding community health education	Health workers	Community meetings were held. VHTs/or male partner ANC/PMTCT access clubs supported in mobilizing communities.	20 months	Twice every month	Pregnant women (and other women of child bearing age) and men
**Health facility based interventions**					
Capacity building for health workers	Health workers	Training on, followed by mentorship comprehensive PMTCT	5 days training. Mentorship took place on quarterly basis for 21 months	5 days training. Mentorship took place on quarterly basis	Health workers in the supported facility
Provision of delivery kits (mama-kits)		Delivery kits were procured and supplied to health facilities. Some items in the kits were then used by health workers to deliver women in health facility. The other items in the delivery kits were handed to mothers after delivery.	10 months	3 times	Pregnant women delivering in the health facility
Provision of HIV testing kits	Project team	Testing-kits were procured by the project and supplied to the health facility as buffer stocks. The kits were used for testing mothers and their spouses during ANC attendance.	These were provided whenever talk-outs were reported. The project therefore ensured that HIV test kits were available for the entire period (21 months).	These were provided whenever talk-outs were reported	Pregnant women and their spouses
HIV counselling and testing during ANC	Health workers	HIV counselling and testing of pregnant women and their spouses during ANC attendance at health facility. Their HIV test results were also given to them at the same point. This was done as part of the ANC services package.	21 months	At least twice every week	Pregnant women and their spouses attending ANC

#### ***Health workers’ capacity building***

Health facility capacity building was done through training of health workers with ongoing mentorship, and provision of basic supplies such as delivery kits (also known as mama-kits), drugs, and HIV test kits. The mama-kit includes basic delivery supplies: cotton wool (200 g), hydrophilic gauze, sterile surgical gloves, mackintosh (rubber/polythene sheet), washing soap (600 g), surgical blades, umbilical-cord tie and tetracycline eye ointment (for the new born baby). These kits were also supplied to facilities by Ministry of Health (MoH) but stock outs were experienced by the health centre. Stock out of delivery supplies and the requirement for “poor” mothers to buy these supplies has been documented as one of the hindrances to health facility deliveries. In order to attract mothers to deliver at the health facility; baby soap, a baby towel and shawl were also added into the mama-kit. The modified delivery kits (with additional baby items) were given to pregnant women when they turned up to deliver at the health facility since anecdotal information suggested that some mothers used these kits to deliver elsewhere, within the communities, and their distribution at ANC may be viewed as encouraging women to deliver at home. Mama-kits were given free of charge.

#### ***Community mobilization and sensitization effort***

The community interventions included activities such as music, dance and drama (MDD), use of village health teams (VHT) for community sensitization, and establishment of male partner access clubs to sensitize and mobilize communities. The key messages to the community included benefits of ANC and skilled delivery attendance as well as male partner involvement in ANC. Community mobilization and sensitization was carried out using existing community structures; VHT are the frontline community workers and are recommended by the MoH for community education and health promotion for all diseases and have been used to delivery other health interventions in Uganda [23].

Sixty VHT members were identified and trained. These included both males and females. Selection of the VHT members to support these program interventions was done in close consultation with the district and health facility workers. The trained VHTs were involved in community sensitization and mobilization of pregnant mothers (and their male partners) for ANC attendance and health facility deliveries under the care of skilled birth attendants. The training was based on the national (MoH) 5-day training curriculum. Each VHT member was given a bicycle to facilitate movement within the community. Information Education and Communication (IEC) materials (charts) were designed and provided to VHTs to act as job aides. In addition to community sensitization and mobilization, VHTs were involved in tracking mothers who missed ANC visits and encouraging them to deliver in the health facility. Monthly performance review meetings attended by VHTs and health workers were conducted to improve performance and the relationship between VHTs and health workers, and also acted as supervisory encounter by health workers.

#### ***Male partner ANC/prevention of mother to child transmission of HIV (PMTCT) access clubs***

Two male partner PMTCT access clubs were formed and engaged. These clubs comprised selected men from the community, who were viewed as role models in participating in ANC with their pregnant wives and ensuring that their wives delivered in the health facility. Male partner access clubs were facilitated to conduct two 'male dialogue’ meetings in each month with other men in the communities. The aim of these meetings was to promote male partner involvement in ANC and skilled attended deliveries by their pregnant wives.

#### ***Music, dance and drama (MDD) groups***

Two MDD groups were established, trained and provided with the necessary costumes. Each of the two groups was facilitated to conduct two community mobilization and sensitization performances monthly. These groups focused on promoting ANC and skilled deliveries attendance as well as PMTCT services.

#### ***HIV counseling and testing during ANC***

Pregnant women and their husbands who turned up at health facility for ANC were counseled, tested and they received their HIV results together (as couples) on their first ANC visit. Pregnant women who turned up for ANC without their husbands were also counseled, tested and received their HIV results as individuals and encouraged to come with their partners on subsequent visits.

### Data collection procedure

All data which also included the estimated (expected) number of pregnant women in the first trimester were abstracted from the Health Information Management System (HIMS) data collection and reporting tools as well as registers of MOH that are used routinely by the health workers to capture patient care and treatment data. These included: the ANC register, delivery and birth register as well as quarterly and annual HMIS reports. Based on the population demographics, the health facility with technical support from the district health office and MoH set the annual targets on expected pregnancies. These facility level targets contributed to the district and national annual targets [24,25]. The data that was used for this paper was collected over a period of 21 months (from January, 2010 to September, 2011); during implementation of interventions. In order to evaluate the outcomes of the project, data for the same indicators was collected for the year preceding the interventions (January to December, 2009). The data was abstracted from the registers using a standardized tool and entered into an excel sheet for storage and analysis.

### Data analysis

Data was analyzed using Excel and Epi Info soft ware. Although data was collected on a monthly basis, during analysis data was collapsed into quarters and years (annually). The variables of interest included: total number of pregnant women attending first ANC visit and this was compared with the estimated annual number of pregnant women in the first trimester who were expected to attend ANC. Other measures included; total number of women attending the fourth ANC, number of pregnant women attending the first ANC visit who were counseled, tested for HIV and received their results (this was compared with the total number of pregnant women attending the first ANC visit rather than the total number of pregnancies in the community), number of male partners of pregnant women attending the first ANC and counseled, tested for HIV and received their results together with their wives (this was compared with number of pregnant women attending the first ANC visit who were counseled, tested for HIV and received their results);first-time ANC pregnant women were expected to be counseled, tested for HIV and given their HIV status results together with their husbands, as couples. The other variable was total number of skilled attended (health facility) deliveries recorded in the facility registers. Pregnant women who attended ANC but did not return to deliver in the health facility were considered to have delivered in the community/at home with the help of unskilled personnel. Using EPINFO StatCal option, we performed a Chi square test for trend and generated Odds Ratios (OR), 95%CI and p-Values to check whether the trends in these indicators were statistically significant. The Confidence Intervals (CI) were computed using the usual Chi-square in EPINFO.

### Ethical approval

This manuscript was based on routine program data analysis for purposes of program monitoring and evaluation. The data that were analyzed had no identifiers for any of the individuals that were served and was exempt from ethical approval.

## Results

### Trends of ANC attendance by pregnant women

**Figure 1:**
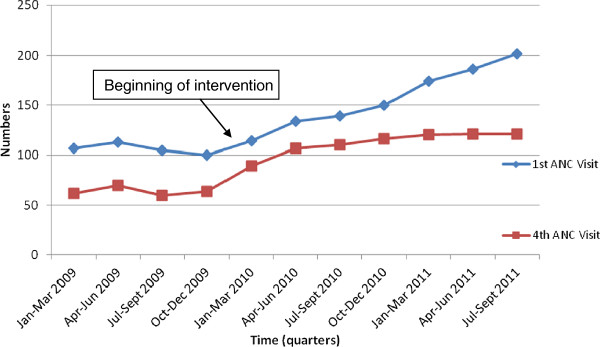
Trends in ANC attendance by pregnant women.

Generally the total annual first ANC attendance increased from the year 2009 to 2011. In 2009, 56.0% (425/753) of the expected women attended ANC compared to 69.0% (537/778) in 2010 (OR 1.72; 95% CI 1.39 – 2.12; p < 0.001) and 93.7% (562/600) in 2011 (OR 11.41; 95% CI 7.97 – 16.34; p < 0.001). Refer to Table 2. More specifically, in 2009, there was no significant change in the number of mothers attending both first and fourth ANC visits. The numbers however increased from 114 women in the first quarter of 2010 to 202 in the third quarter of 2011. This trend continued steadily for the new ANC attendees up to the third quarter of 2011. A rise in fourth ANC visit was registered in 2010, from 89 to 107 in the first and second quarters respectively of 2010, with a slower rise from the third quarter of 2010 to third quarter of 2011. In all the quarters from 2009 to 2011 fewer women attended 4th ANC visit compared to those who attended 1st ANC visit (Figure 1).

**Table 2:** ANC attendance by pregnant women before and during implementation of systems strengthening interventions

**Year**	**Expected ANC attendees**	**Attended ANC**	**OR**	**95% CI**	**p-value**	**Chi square for trend**
2009	753	425	1.00			221.11
2010	778	537	1.72	1.39-2.12	p < 0.001	
2011	600	562	11.41	7.97-16.34	p < 0.001	
Total	2131	1524				

Data Source for expected ANC attendees: District annual reports.

### Trends of HIV counseling and testing of pregnant women during ANC attendance

**Figure 2:**
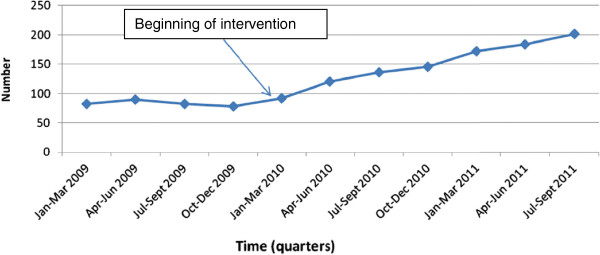
Trends in HIV counselling and testing of pregnant women during ANC attendance.

The number of pregnant women who were counselled, tested and received their results for HIV was relatively stable in 2009 (pre-intervention). During this period, 82 (76.6%) pregnant women in the first quarter, 90 (79.6%) in the second quarter, 83 (79.0%) in the third quarter and 78 (78.0%) in the fourth quarter were counselled and tested. However, a steady rise was registered from 92 (80.7%) in the first quarter to 146 (97.3%) in the fourth quarter of 2010 (OR 3.40; 95% CI 2.31 – 4.99; p < 0.001) and then to 201 (99.5%) pregnant women in the third quarter of 2011 (OR 32.94; 95% CI 13.27 – 81.77; p < 0.001) (Figure 2 and Table 3).

**Table 3:** Uptake of HIV counselling and testing by pregnant women during ANC attendance before and during implementation of systems strengthening interventions

**Year**	**ANC attendance (to be tested)**	**Tested**	**Not tested**	**OR**	**95% CI**	**p-value**	**Chi square for trend**
2009	425	328	97	1.00			130.13
2010	537	494	43	3.40	2.31-4.99	p < 0.001	
2011	562	557	5	32.94	13.27-81.77	p < 0.001	
Total	1524	1379	145				

Data Source: ANC registers.

### Trends of male partner counseling and testing in ANC with their pregnant wives

**Figure 3:**
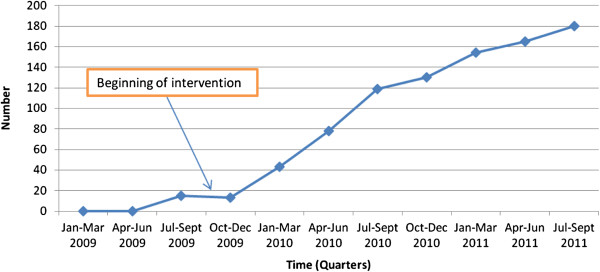
Trends in male partner counselling and testing for HIV during ANC attendance with their pregnant wives.

The number of male partners undergoing HIV counseling and testing together with their pregnant wives during ANC was computed. In the first two quarters of 2009, male ANC attendance was at zero. The number of male partner ANC attendance steadily rose from 13 (16.7%) in the fourth quarter of 2009 to 130 (89%) in the fourth quarter of 2010 (OR 31.97; 95% CI 20.64 – 49.51; p < 0.001) and then to 180 (89.6%) in the third quarter of 2011 (OR 92.18; 95% 57.43 – 147.96; p < 0.001) (Figure 3 and Table 4).

**Table 4:** Male partners counselled and tested together with their pregnant wives during ANC attendance before and during implementation of systems strengthening interventions

**Year**	**Pregnant women tested**	**Male partners tested**	**Male partners not tested**	**OR**	**95% CI**	**p-value**	**Chi square for trend**
2009	328	28	300	1.00			536.51
2010	494	370	124	31.97	20.64-49.51	p < 0.001	
2011	557	499	58	92.18	57.43-147.96	p < 0.001	
Total	1379	897	482				

Data Source: ANC registers.

### Trends of health facility deliveries and provision of mama-kits

**Figure 4:**
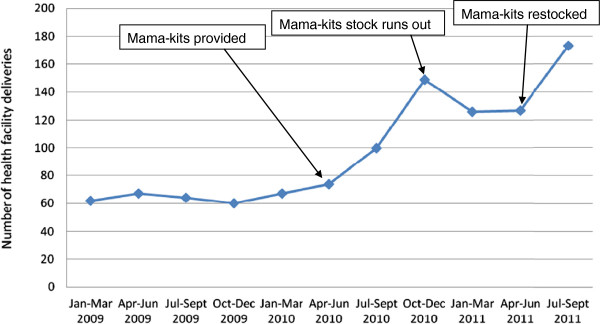
Trends in health facility deliveries.

From the first quarter of 2009 to the second quarter of 2010 the number of pregnant women delivering in the health facility remained relatively stable. The number of pregnant women delivering in the health facility did not change much before distribution of mama kits, at 67 (58.8%) in the first quarter and74 (55.2%) in the second quarter of 2010. However, there was an increase to 100 (71.9%) and 149 (99.3%) in the third and fourth quarters of 2010 respectively. Mama-kits were provided at the end of second quarter of 2010 and lasted up to the fourth quarter of 2010. In the first and second quarter of 2011 after the mama-kit stock had run out, the number of pregnant women delivering in the health facility dropped to 126 (72.4%) and 127 (68.3%) respectively but remained higher than the period before mama-kits were provided. When mama-kits were re-stocked at the beginning of the third quarter of 2011 the number of pregnant women delivering in the health facility increased again, to 173 (85.6%) in the third quarter of 2011 (Figure 4).

## Discussion

This evaluation demonstrates a rise in key ANC and delivery indicators following community mobilization and health facility capacity strengthening. ANC attendance, up take of HIV counseling and testing by pregnant women and their partners as well as health facility deliveries increased with program interventions.

### Trends of ANC attendance by pregnant women

ANC attendance provides an opportunity for assessment of women’s health and planning for delivery and is an important level in the improvement of maternal and child health outcomes [6-8]. The rising first ANC attendance in this evaluation is consistent with those in another study conducted in a rural district in Tanzania, which showed increased ANC booking following community engagement [15]. The community engagements in these program interventions also addressed cultural beliefs and barriers which have been shown to influence ANC attendance [26]. In this evaluation, the fourth ANC attendance was however not impacted much suggesting that more efforts are required for communities to appreciate the need for follow-up visits, and facilities to ensure retention of women after the first visit [27].

### Uptake of HIV counseling and testing by pregnant women

The uptake of HIV testing by pregnant women rose during the implementation of the interventions and can be attributed to community mobilisation as well as emphasizing testing at first ANC visit. Deferring HIV testing to subsequent visits, which sometimes happens in ANC programs, is a missed opportunity as several women will not return after the first ANC visit, as demonstrated in this evaluation and other studies [28]. Ensuring availability of supplies like test kits at the facility may have also contributed to the increased uptake of HIV testing by women and their partners [29].

### Male partner involvement in ANC

These findings show a rapid increase in male partner involvement in ANC and male partner uptake of HIV testing, an area that is very crucial in the delivery of maternal and child health services. This is a very important finding given the continued challenges that programs have faced with promoting male involvement in various services including family planning, ANC, delivery and PMTCT. For example, in Uganda men have been observed to be particularly important in ensuring that their pregnant women attend skilled deliveries [30]. The male partner access clubs and VHTs recruited men who had been engaged in ANC and whose wives had delivered at health facilities, and these may have acted as 'change agents’ [18]. These kind of interventions may also overcome the cultural barriers that influence ANC attendance within communities [22].

### Facility based deliveries

Skilled delivery care is crucial in saving the lives of mothers and their infants [31]. Provision of delivery supplies had a significant and fairly sustained impact in terms of increasing health facility deliveries; whether or not these supplies are delivered as mama-kits or other form, ensuring availability of delivery supplies is an important intervention in increasing facility based deliveries. The additional items in the mama kits for the babies may have also acted as an added incentive for facility based deliveries, an area that may require further scrutiny, since it was implemented alongside a myriad of other interventions. Whereas other interventions such as community mobilization and male partner involvement may have contributed, there was a clear link between the availability of delivery kits and facility based deliveries. As documented in various studies, the availability of mama-kits could have addressed the challenges associated with poor families procuring delivery items as is often required in times of stock outs at facilities, [32-34].

We cannot fully explain the sustained facility deliveries after the mama-kits stocks ran out. However, it is possible that availability of mama-kits attracted pregnant women to deliver in the health facility and once they got to the facilities, the women got exposed to other interventions (e.g. health education) that may have influenced the development of positive attitudes towards delivering in the health facility. The interventions aimed at improving quality of health services (e.g. training of providers and availability of other supplies) may have also instilled confidence and sustained the facility deliveries despite the absence of mama kits.

The project team engaged different stakeholders including other non-governmental organizations and the district local government to appreciate the crucial and significant role that mama-kits and other interventions in this project play in promoting ANC attendance by women and their partners, and skilled attended deliveries. This was done with a view of encouraging other stakeholders to take up these interventions and ensure continuity.

## Conclusions

Concurrent implementation of community mobilization and facility capacity strengthening increased ANC attendance and health facility deliveries as well as male partner involvement. Specifically, provision of free delivery kits to mothers who delivered at the health facility significantly increased skilled attended delivery. More women attended first ANC. However, few mothers returned for up to four ANC visits. Programs that aim at increasing uptake of ANC and attended deliveries should simultaneously address demand and quality gaps at facilities. More efforts are required to enhance attendance of ANC up to four times; until this is realized, key ANC interventions should be tied to the first ANC visit to ensure that they are delivered.

### Study limitations

This evaluation has several limitations which we acknowledge. We could not completely rule out the contributions by other players in the attainment of the health outcomes described in this paper. However given that ChildFund Uganda was the main implementing agency of these interventions in that region at the time of the evaluation, the results registered can to a great extent be safely attributable to these interventions.

The absence of information on characteristics of the study population and pregnancy history of the mothers as well as male partner variables limits the analysis for the women-specific factors affecting uptake of these services. The authors cannot guarantee the accuracy of estimated/expected annual numbers of pregnant women in the first trimester, which may compromise the accuracy of the proportions of the women reached. Additionally, the data on pregnancy outcomes was not included in the analysis, and the outcomes are limited to the service uptake indicators. Also, this analysis only tracked uptake of HIV testing and not the other services in the ANC package. The exact cost of mama-kits and other interventions were not computed given a number of indirect costs associated their delivery. Finally our paper does not present the number/percentage of pregnant women who delivered at home (under unskilled personnel). However, the evaluation is still important as it demonstrates increased first ANC attendance, facility based deliveries, and male involvement, which are important avenues for increasing all ANC and facility delivery services.

## Competing interests

The authors declare that they have no competing interests.

## Authors’ contributions

ME: participated in the design and implementation of the program interventions, data collection and analysis, conceived and wrote the first draft of the manuscript; RKW: participated in the design of the manuscript, data interpretation drafting and reviewing of the manuscript; SM: participated in program design and drafting of the manuscript; GO: participated in program design, drafting of the manuscript; RI: participated in program design and reviewed the manuscript; AO: participated in drafting and reviewing the manuscript, NMT: participated in the conception, data interpretation drafting and reviewing of manuscript. All authors reviewed and approved the final version of the manuscript.

## Pre-publication history

The pre-publication history for this paper can be accessed here:

http://www.biomedcentral.com/1471-2393/13/189/prepub
